# Eupatilin Suppresses OVA-Induced Asthma by Inhibiting NF-κB and MAPK and Activating Nrf2 Signaling Pathways in Mice

**DOI:** 10.3390/ijms23031582

**Published:** 2022-01-29

**Authors:** Donghui Bai, Tianxiao Sun, Fang Lu, Yancheng Shen, Yan Zhang, Bo Zhang, Guangli Yu, Haihua Li, Jiejie Hao

**Affiliations:** 1 Key Laboratory of Marine Drugs, Ministry of Education, School of Medicine and Pharmacy, Ocean University of China, Qingdao 266003, China; bdh2126@stu.ouc.edu.cn (D.B.); suntianxiao@stu.ouc.edu.cn (T.S.); 21200831158@stu.ouc.edu.cn (F.L.); shenyancheng2021@sibs.ac.cn (Y.S.); 21210813099@stu.ouc.edu.cn (Y.Z.); pajkklxfzhang@163.com (B.Z.); glyu@ouc.edu.cn (G.Y.); 2 Shandong Provincial Key Laboratory of Glycoscience and Glycotechnology, School of Medicine and Pharmacy, Ocean University of China, Qingdao 266003, China; 3 Laboratory for Marine Drugs and Bioproducts, Pilot National Laboratory for Marine Science and Technology (Qingdao), Qingdao 266237, China

**Keywords:** asthma, eupatilin, NF-κB, MAPK, Nrf2

## Abstract

To investigate the effect of eupatilin in asthma treatment, we evaluated its therapeutic effect and related signal transduction in OVA-induced asthmatic mice and LPS-stimulated RAW264.7 cells. The BALF was tested for changes in lung inflammatory cells. Th2 cytokines in the BALF and OVA-IgE in the serum were measured by ELISA. H&E and PAS staining were used to evaluate histopathological changes in mouse lungs. The key proteins NF-κB, MAPK, and Nrf2 in lung tissues were quantitatively analyzed by Western blotting. Finally, we evaluated the effect of eupatilin on cytokines and related protein expression in LPS-stimulated RAW 264.7 cells in vitro. In OVA-induced asthmatic mice, eupatilin reduced the numbers of inflammatory cells, especially neutrophils and eosinophils. Eupatilin also decreased the levels of IL-5, IL-13 in the BALF and OVA-IgE in the serum. Furthermore, eupatilin inhibited the activation of NF-κB and MAPK pathways and increased the expression of Nrf2 in OVA-induced asthmatic mice. In vitro, eupatilin significantly reduced LPS-stimulated NO, IL-6, and ROS production. Additionally, the NF-κB, MAPK, and Nrf2 protein expression in LPS-stimulated RAW264.7 cells was consistent with that in OVA-induced asthmatic lung tissues. In summary, eupatilin attenuated OVA-induced asthma by regulating NF-κB, MAPK, and Nrf2 signaling pathways. These results suggest the utility of eupatilin as an anti-inflammatory drug for asthma treatment.

## 1. Introduction

Asthma is a common chronic respiratory inflammatory disease [1]. It is characterized by airway hyperresponsiveness, allergen-specific IgE secretion, mucus hypersecretion, and airway inflammation [2,3]. In recent years, the incidence of asthma has increased due to increases in indoor dust, pollen, toxic particles, environmental pollutants, and other allergens [4]. It is estimated that 241 million people worldwide suffer from asthma, with 1000 people dying every day [5]. At present, asthma cannot be cured but can only be controlled by medicine, which brings serious physical and psychological damage to patients.

Previous studies have suggested that the imbalance between Th1 and Th2 cells is the major cause of asthma [6]. Th2 cells play a vital role in asthmatic progression [7,8]. Activated Th2 cells oversecrete the cytokines IL-4, IL-5, and IL-13, promoting airway inflammation and remodeling [9,10]. Among these cytokines, IL-5 is central to the asthmatic phenotype, as it can affect the growth, survival, differentiation, and recruitment of eosinophils [11,12]. IL-13 can induce eosinophils to infiltrate lung tissue and directly cause airway inflammation and airway hyperresponsiveness [13,14]. Thus, antagonizing the action of Th2 cytokines represents one of the major therapeutic strategies in the treatment of asthma. In addition, the causes of many problems related to asthma remain unclear, so investigations into the pathogenesis of asthma and identification of new therapeutic targets are urgently needed.

In addition to related cytokines, a host of signaling pathways are known to be involved in the pathophysiology process of asthma, including NF-κB, MAPK, and Nrf2 signaling pathways. NF-κB is a transcription factor that can regulate the production of many cytokines and play a central role in modulating inflammatory and immune responses [15,16]. NF-κB is continuously activated in allergic asthma, and NF-κB inhibition can significantly alleviate ovalbumin (OVA)-induced asthma [17]. Furthermore, it has been confirmed that activation of the mitogen-activated protein kinase (MAPK) pathway is closely associated with airway inflammation [15]. The MAPK family is composed of p38 MAPK, Erk, and JNK. It has been clearly reported that the levels of phosphorylated p38 MAPK, Erk, and JNK are significantly elevated in the airways of patients with allergic asthma and that this increased expression correlates with the severity of asthma [18]. In addition, nuclear factor-erythroid 2-related factor 2 (Nrf2), which is associated with transcriptional activation of the antioxidant response element (ARE) gene, is beneficial in asthma, acting through anti-inflammatory mechanisms [19]. In summary, NF-κB, MAPK, and Nrf2 pathways have become key targets in the treatment of asthma. Therefore, inhibiting NF-κB and MAPK family members while enhancing Nrf2 pathway activity may be an effective approach for preventing or treating asthma.

**Figure 1:**
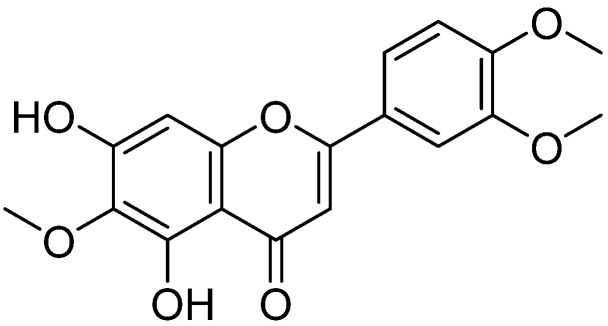
Chemical structure of eupatilin.

It has been reported that many traditional Chinese herbs are effective in reducing asthma symptoms in both humans and animals [20]. Eupatilin [2-(3,4-dimethoxyphenyl)-5,7-dihydroxy-6-methoxychromen-4-one] (Figure 1), a pharmacologically active flavone extracted from *Artemisia argyi*, has a variety of pharmacological activities, including anti-inflammatory, anticancer, antioxidant, antiallergic, cardioprotective, and neuroprotective activities [21,22]. Eupatilin suppresses inflammatory responses by inhibiting the NF-κB signaling pathway and reducing the lipopolysaccharide (LPS)-stimulated production of inflammatory cytokines [23]. Eupatilin has also been found to inhibit TNF-α-induced eosinophil migration [24]. In addition, it has been shown that eupatilin can inhibit allergic inflammatory reactions both in vitro and in vivo, suggesting that eupatilin may be used to treat inflammatory diseases associated with allergic disorders [25,26]. Currently, there are very few studies on eupatilin in lung diseases in mice, and the only report is that eupatilin could attenuate acute lung injury in mice by inhibiting inflammation and oxidative stress [27]. However, the therapeutic activity of eupatilin against asthma in vivo and its associated mechanism have not been studied. In the present study, we first investigated the anti-asthmatic activity and possible mechanism of eupatilin in OVA-induced asthmatic mice and verified it in LPS-stimulated RAW264.7 cells.

## 2. Results

### 2.1. Effect of Eupatilin on Inflammatory Cells in the Bronchoalveolar Lavage Fluid (BALF)

**Figure 2:**
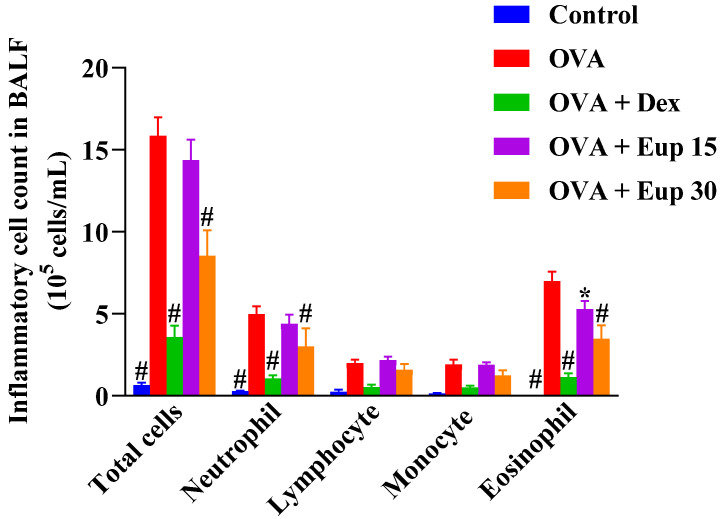
Effect of eupatilin on OVA-induced inflammatory cell count in the BALF. Twenty-four hours after the last challenge, the total inflammatory cells, neutrophils, lymphocytes, monocytes, and eosinophils in the BALF were counted. Data represent the mean ± SEM (*n* = 7). * *p* < 0.05, # *p* < 0.0001 vs. OVA group.

In this study, the effect of eupatilin on the profile of inflammatory cells in the BALF was detected. As shown in Figure 2, compared with the control group, the OVA group showed significantly increased numbers of total inflammatory cells, neutrophils, lymphocytes, monocytes, and eosinophils in the BALF. Eupatilin administration could reduce the number of inflammatory cells in the BALF, especially neutrophils and eosinophils, to varying degrees, indicating that eupatilin could alleviate inflammatory cell infiltration in the lungs of asthmatic mice.

### 2.2. Eupatilin Reduces OVA-Induced Th2 Cytokine Levels in the BALF and OVA-IgE Levels in the Serum

**Figure 3:**
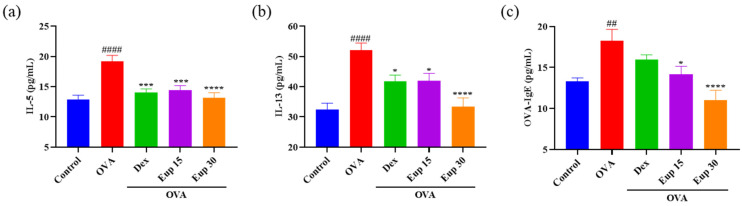
Effect of eupatilin on OVA-induced Th2 cytokines in the BALF and OVA-IgE levels in the serum were detected by ELISA. (**a**) BALF IL-5 levels. (**b**) BALF IL-13 levels. (**c**) Serum OVA-IgE levels. Data represent the mean ± SEM (*n* = 7). ## *p* < 0.001, #### *p* < 0.0001 vs. control group; * *p* < 0.05, *** *p* < 0.001, **** *p* < 0.0001 vs. OVA group.

In asthma, inflammatory responses are closely associated with the activation of Th2 cells [28]. Th2 cytokines can activate eosinophils and induce B cells to produce IgE [29]. Compared with control treatment, OVA sensitization and challenge significantly increased the production of IL-5, IL-13, and OVA-IgE (Figure 3). However, eupatilin administration inhibited the increase of these cytokines in a dose-dependent manner, and it was also significantly more effective than dexamethasone at a dose of 30 mg/kg.

### 2.3. Effect of Eupatilin on Lung Histological Changes in Asthmatic Mice

**Figure 4:**
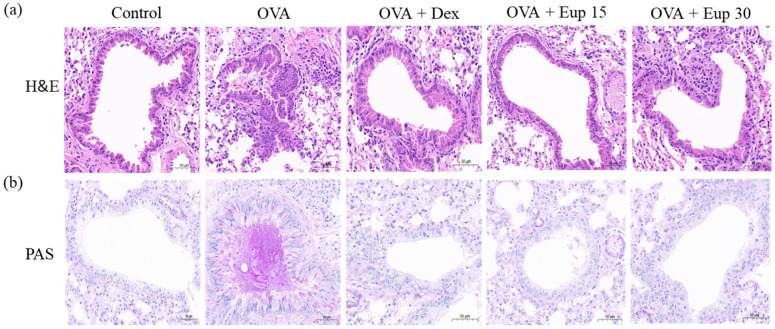
Effect of eupatilin on lung histological changes in OVA-induced asthmatic mice. (**a**) H&E staining was used to detect inflammatory cell infiltration. (**b**) PAS staining was used to detect the production of mucus around the airways. 200× magnification; scale bar: 50 μm.

Inflammatory cell infiltration and mucus secretion are the key features of allergic asthma. Hematoxylin and eosin (H&E) and periodic acid-Schiff (PAS) staining analyses were used to detect histological changes in lung tissues. As shown in Figure 4a, lung tissues from the OVA group exhibited significant inflammatory cell infiltration. Additionally, PAS staining showed that the mice in the OVA group overproduced mucus (Figure 4b). However, eupatilin treatment could significantly improve the inflammatory cell infiltration induced by OVA and remarkably inhibit mucus hypersecretion, which were comparable with the effect of dexamethasone.

### 2.4. Effect of Eupatilin on NF-κB, MAPK and Nrf2 Signaling Pathways in Asthmatic Mice

**Figure 5:**
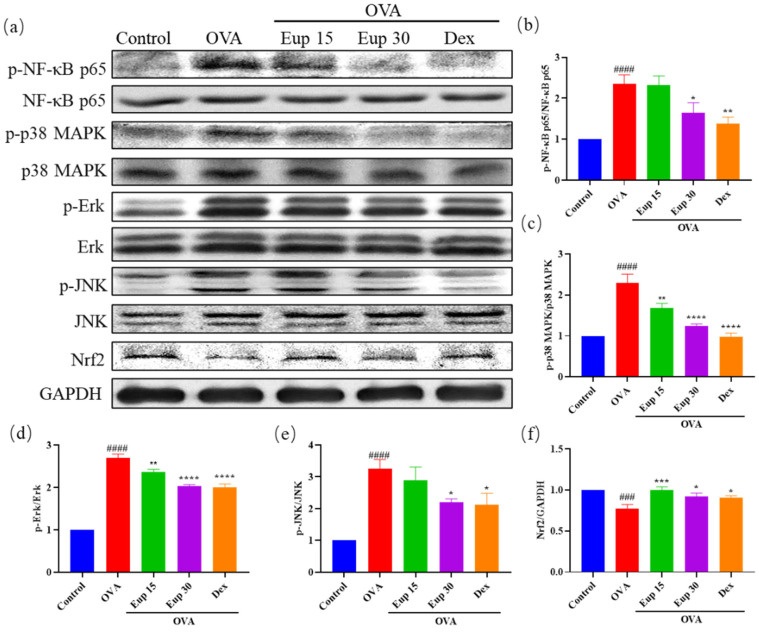
Effect of eupatilin on NF-κB, MAPK, and Nrf2 signaling pathways in OVA-induced asthmatic mice. (**a**) Western blotting analyses of NF-κB p65, p-NF-κB p65, p38 MAPK, p-p38 MAPK, Erk, p-Erk, JNK, p-JNK, and Nrf2 protein expression in lung tissues. (**b**) Quantification of the p-NF-κB p65/NF-κB p65 ratio. (**c**) Quantification of the p-p38 MAPK/p38 MAPK ratio. (**d**) Quantification of the p-Erk/Erk ratio. (**e**) Quantification of the p-JNK/JNK ratio. (**f**) Quantification of the Nrf2/GAPDH ratio. Data represent the mean ± SEM (*n* = 3). ### *p* < 0.001, #### *p* < 0.0001 vs. control group; * *p* < 0.05, ** *p* < 0.01, *** *p* < 0.001, **** *p* < 0.0001 vs. OVA group.

NF-κB and MAPK are key targets associated with inflammation and play an important role in asthma inflammation. To explore the effect of eupatilin on NF-κB and MAPK signaling pathways, the protein levels of p-NF-κB p65, p-p38 MAPK, p-Erk, and p-JNK were detected by Western blotting (Figure 5a). Compared with OVA induction alone, eupatilin treatment significantly inhibited the OVA-induced phosphorylation of NF-κB (Figure 5b). Furthermore, we observed that the levels of phosphorylated MAPK family members (p38 MAPK, Erk, and JNK) were significantly enhanced in the OVA group, whereas eupatilin treatment significantly inhibited the activation of p38 MAPK, Erk, and JNK (Figure 5c–e).

Inflammation itself leads to oxidative stress in the airways and lungs, further exacerbating the inflammatory response [30]. Therefore, the protein expression levels of Nrf2 in lung tissues were evaluated. The results show that OVA administration significantly inhibited Nrf2 expression, while Nrf2 expression levels were significantly increased after eupatilin administration, and the effect was comparable with that of dexamethasone (Figure 5f). These results suggest that eupatilin could alleviate the inflammatory reactions in asthma by affecting NF-κB, MAPK, and Nrf2 signaling pathways.

### 2.5. Effect of Eupatilin on Inflammatory Cytokines in RAW264.7 Cells

**Figure 6:**
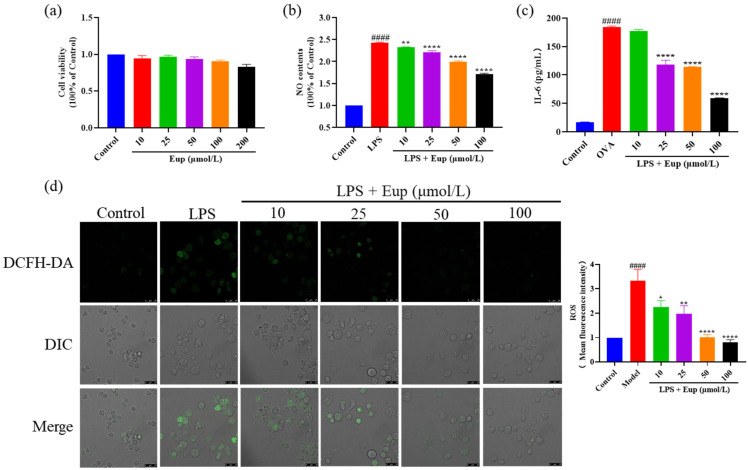
Effect of eupatilin on inflammatory cytokines in LPS-stimulated RAW264.7 cells. RAW264.7 cells were induced with 1 µg/mL LPS and treated with various concentrations (10, 25, 50, and 100 µmol/L) of eupatilin for 24 h. (**a**) Cell viability after treatment with different concentrations of eupatilin for 24 h. (**b**) NO levels in cell supernatants after LPS and eupatilin treatment. (**c**) IL-6 levels in cell supernatants after LPS and eupatilin treatment. (**d**) ROS levels in RAW264.7 cells treated with LPS and eupatilin. 630× magnification; scale bar: 25 μm. Data represent the mean ± SEM (*n* = 4). #### *p* < 0.0001 vs. control group; * *p* < 0.05, ** *p* < 0.01, **** *p* < 0.0001 vs. LPS group.

Previous studies have revealed that alveolar macrophages may play a central role in promoting airway inflammation [31]. After treatment with different concentrations of eupatilin, the observed cell viability was not significantly different from that of the control group, indicating that eupatilin had no significant cytotoxicity up to a dose of 200 μmol/L (Figure 6a). Compared with the LPS group, eupatilin significantly reduced the release of NO and IL-6, with eupatilin acting in a dose-dependent manner (Figure 6b,c). Furthermore, LPS significantly induced ROS production in RAW264.7 cells, while eupatilin significantly inhibited ROS production (Figure 6d). These results suggest that eupatilin had anti-inflammatory activity in LPS-stimulated RAW264.7 cells.

### 2.6. Effect of Eupatilin on NF-κB, MAPK, and Nrf2 Signaling Pathways in RAW264.7 Cells

**Figure 7:**
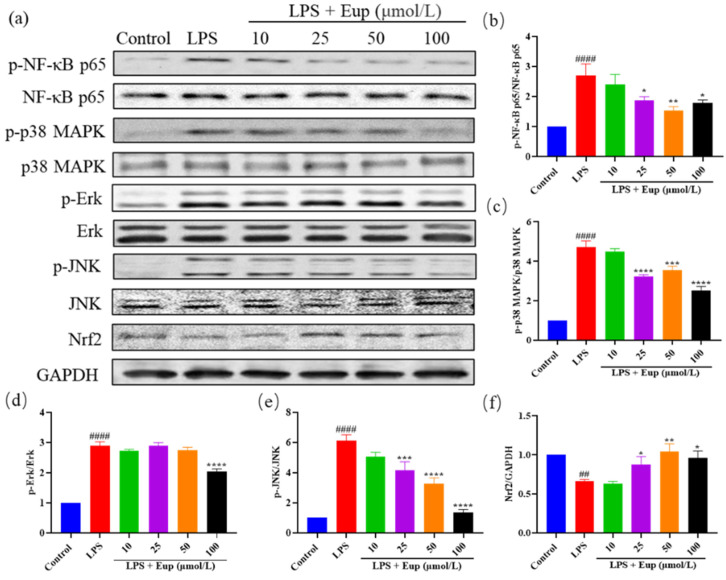
Effect of eupatilin on NF-κB, MAPK, and Nrf2 signaling pathways in LPS-stimulated RAW264.7 cells. After RAW264.7 cells were treated with LPS and eupatilin for 24 h, intracellular proteins were extracted for subsequent Western blotting analysis. (**a**) Western blotting analyses of NF-κB p65, p-NF-κB p65, p38 MAPK, p-p38 MAPK, Erk, p-Erk, JNK, p-JNK, and Nrf2 protein expression in LPS-stimulated RAW264.7 cells. (**b**) Quantification of the p-NF-κB p65/NF-κB p65 ratio. (**c**) Quantification of the p-p38 MAPK/p38 MAPK ratio. (**d**) Quantification of the p-Erk/Erk ratio. (**e**) Quantification of the p-JNK/JNK ratio. (**f**) Quantification of the Nrf2/GAPDH ratio. Data represent the mean ± SEM (*n* = 3). ## *p* < 0.01, #### *p* < 0.0001 vs. control group; * *p* < 0.05, ** *p* < 0.01, *** *p* < 0.001, **** *p* < 0.0001 vs. LPS group.

To further verify the therapeutic target of eupatilin in asthma, we detected the protein expression of p-NF-κB p65, p-MAPK, and Nrf2 in LPS-stimulated RAW264.7 cells (Figure 7a). The levels of phosphorylated NF-κB p65, p38 MAPK, Erk, and JNK in the LPS group were significantly increased, while eupatilin significantly inhibited the phosphorylation of these proteins (Figure 7b–e). In addition, compared with the LPS group, the eupatilin group showed significantly elevated expression of Nrf2 (Figure 7f). The protein expression trends in LPS-stimulated RAW264.7 cells were consistent with those in mouse lung tissues, further indicating that eupatilin exerted its anti-inflammatory effects in asthma through effects on NF-κB, MAPK, and Nrf2 signaling pathways.

## 3. Discussion

Asthma is a recurrent chronic airway inflammatory disease involving multiple inflammatory cells and mediators [32,33]. Glucocorticoids are considered to be the best choice to treat asthma. However, they have limited efficacy and can cause various adverse reactions. Therefore, there is an urgent need to find a new, safer, and more effective drug to treat asthma [34,35]. Pharmacological and phytochemical studies have identified many potential anti-inflammatory ingredients, especially options derived from traditional Chinese medicine, so traditional Chinese herbal medicine is becoming an important source of active drugs [36]. Eupatilin has good anti-inflammatory activity, but its therapeutic activity in asthma has not been explored. Our results confirm that eupatilin attenuated OVA-induced asthma by inhibiting NF-κB and MAPK and activating Nrf2 signaling pathways.

The inflammatory responses in asthma involve the excessive production of IgE by B cells, release of inflammatory cytokines, and infiltration of inflammatory cells [37]. A variety of inflammatory cells are involved in airway inflammation, such as macrophages, eosinophils, lymphocytes, and neutrophils [38,39]. Among them, eosinophils are the main contributors to allergic inflammation and are involved in the induction of airway hyperresponsiveness and remodeling in asthma [40,41]. In this study, our results show that eupatilin could reduce the number of inflammatory cells in the BALF, especially neutrophils and eosinophils. Th2 cells play a key role in the pathogenesis of allergic asthma inflammation [42,43]. Th2 cytokines, including IL-5 and IL-13, are involved in eosinophil accumulation, mucus hypersecretion, and allergen-specific IgE secretion [44]. In this study, OVA sensitization and challenge could significantly increase the production of IL-5, IL-13, and OVA-IgE. Eupatilin administration inhibited the increase of IL-5, IL-13, and OVA-IgE in a dose-dependent manner, and the effects were better than dexamethasone. In addition, eupatilin treatment could significantly improve inflammatory cell infiltration induced by OVA and could remarkably inhibit mucus hypersecretion, which were comparable with the effect of dexamethasone. These results suggest that eupatilin could play an anti-asthmatic role by reducing inflammatory cell infiltration.

Next, we further explored the molecular mechanisms of eupatilin on OVA-induced asthma. Many studies have shown that NF-κB, MAPK, and Nrf2 are important targets in the treatment of asthma. NF-κB, one of the most studied transcription factors, plays an important role in the mechanisms of various acute and chronic inflammatory conditions, including asthma [45,46,47]. In general, NF-κB binds to IκB in the cytoplasm in an inactive state. When IκB is phosphorylated by the IκB kinase complex (IKK), NF-κB is released and transported to the nucleus, triggering multiple intracellular inflammatory signaling pathways [48,49]. The activation of NF-κB promotes the expression of inflammatory factors, such as NO, COX-2, and IL-6, and the secretion of Th2 cytokines (IL-4, IL-5, and IL-13) in allergic airway inflammation [50]. MAPKs (p38 MAPK, Erk, and JNK) can regulate cellular responses to external stimuli and various cellular activities such as apoptosis, differentiation, inflammation, and gene expression [51,52]. Inhibitors targeting MAPKs have been developed to treat a variety of inflammatory diseases [53]. Nrf2 is one of the most important antioxidative stress proteins. When stimulated, Nrf2 is activated and binds to the ARE, thereby activating the transcription of antioxidant genes [54]. In our results, eupatilin significantly inhibited the phosphorylation of NF-κB, p38 MAPK, Erk, and JNK and increased the expression of Nrf2 in OVA-induced mice. These findings suggest that eupatilin attenuated asthma through NF-κB, MAPK, and Nrf2 signaling pathways.

Macrophages also play an important role in asthma, and the inflammatory factors produced by macrophages, such as IL-6, NO, and ROS, are also important markers of asthma [55,56,57]. NF-κB, MAPK, and Nrf2, as inflammatory signaling pathways, were activated after LPS stimulation of macrophages [58]. Although the mechanism of macrophage stimulation by LPS is very different from the sensitization process observed in asthma, macrophages are also important targets for asthma treatment. In this study, we used LPS stimulation to assess the anti-inflammatory activity and mechanisms of eupatilin. We found that eupatilin reduced NO, IL-6, and ROS levels in RAW264.7 cells and had a relatively good anti-inflammatory effect. Furthermore, eupatilin significantly inhibited the phosphorylation of NF-κB, p38 MAPK, Erk, and JNK and increased the expression of Nrf2 in LPS-stimulated RAW264.7 cells. The protein expression trends in LPS-stimulated RAW264.7 cells were consistent with those in animal lung tissues, further indicating that eupatilin exerted an anti-inflammatory effect in asthma through NF-κB, MAPK, and Nrf2 signaling pathways.

In summary, eupatilin attenuated OVA-induced asthma by regulating NF-κB, MAPK, and Nrf2 signaling pathways. Eupatilin may be a promising therapeutic agent for the treatment of asthma.

## 4. Materials and Methods

### 4.1. Drugs and Reagents

Eupatilin (Eup, purity ≥ 98%) was purchased from Nanjing Spring & Autumn Biological Co., Ltd. (Nanjing, China). Dexamethasone (Dex) was purchased from Beijing Solarbio Science & Technology Co., Ltd. (Beijing, China). Aluminum hydroxide [Al(OH)_3_] was purchased from Thermo Fisher Scientific (Waltham, MA, USA), and OVA was purchased from Sigma Chemical Co. (St. Louis, MO, USA). Mouse IL-6, IL-5, IL-13, and OVA-IgE enzyme-linked immunosorbent assay (ELISA) kits were purchased from Shanghai FANKEW Industrial Co., Ltd. (Shanghai, China). BCA protein detection kit and NO and ROS assay kits were purchased from Beyotime Biotechnology (Shanghai, China). Antibodies against p-NF-κB p65 (3033T), NF-κB p65(8242T), p-p38 MAPK (4511T), p38 MAPK (8690T), p-Erk (4370T), Erk (4695T), p-JNK (4668T), Nrf2 (12721T), and GAPDH (5174T) were obtained from Cell Signaling Technology (CST; Beverly, MA, USA). An antibody against JNK (sc-7345) was purchased from Santa Cruz Biotechnology (Dallas, TX, USA). Fetal bovine serum (FBS) and DMEM medium were purchased from Gibco (Grand Island, NY, USA).

### 4.2. Animals

Female BALB/c mice (6–8 weeks) were obtained from Jinan Pengyue Laboratory Animal Co., Ltd. (Jinan, China). The mice were given plenty of food and water, housed at 25 °C under a 12 h light/dark cycle. Before experimentation, the mice were acclimatized to feeding in the experimental environment for 1 week. All animal experiments were carried out in accordance with the guidelines of the Animal Experiments Ethics Committee of Ocean University of China (OUC-SMP-2021-02-02).

### 4.3. Sensitization and Treatment Protocols

A total of 35 mice were randomly divided into 5 groups with 7 mice in each group: the control group, OVA group, OVA + Dex (1 mg/kg) group, OVA + Eup 15 group, and OVA + Eup 30 group. Dex was used as the positive control drug. The OVA-induced asthmatic model was established as described in a previous study [59]. Briefly, each mouse was sensitized by intraperitoneal injection of 20 μg OVA and 2 mg aluminum hydroxide in 200 μL PBS on days 0, 7, and 14. On days 21, 22, and 23, the OVA-challenged mice were exposed to ultrasonic atomization of 5% OVA for 30 min each day. The mice in the control group were sensitized and challenged with an equal amount of PBS instead of OVA. From day 17 to day 23, mice were treated with eupatilin (15 mg/kg, 30 mg/kg), Dex (1 mg/kg) or an equal amount of 0.9% NaCl solution by intraperitoneal injection. The mice were sacrificed 24 h after the last challenge.

### 4.4. BALF Collection and Leukocyte Counts

Twenty-four hours after the last challenge, the trachea was intubated, and the lungs were washed with 0.7 mL PBS twice to collect the BALF. The BALF was centrifuged at 1500× *g* rpm for 10 min, and the supernatants were collected and stored at –80 °C for cytokine detection. The cell precipitates in the BALF were suspended in 100 µL PBS, and the total numbers of white blood cells, neutrophils, lymphocytes, monocytes, and eosinophils in the BALF were determined with ProCyte Dx^®^ Hematology Analyzer (IDEXX Laboratories Inc., Westbrook, ME, USA).

### 4.5. Determination of IL-5, IL-13 and OVA-IgE Levels

The levels of IL-5 and IL-13 in the BALF and OVA-specific IgE in the serum were determined by ELISA, as described in the corresponding kit instructions.

### 4.6. H&E and PAS Staining

Lung tissues were fixed in 4% paraformaldehyde. Then, the lungs were dehydrated, embedded in paraffin, and sectioned. The sections were stained with H&E or PAS. Finally, the histopathologic changes and mucus content of the lung tissues were examined under a light microscope. Three random fields of each sample were selected at random and photographed under 200× magnification.

### 4.7. Cell Culture and Treatment

The RAW264.7 cell line was obtained from the Cell Bank of the Chinese Academy of Sciences (Shanghai, China) and cultured in Dulbecco’s modified Eagle’s medium (DMEM) supplemented with 10% inactivated fetal bovine serum (FBS) in an atmosphere of 5% CO_2_ at 37 °C.

First, RAW264.7 cells (2 × 10^4^ cells/well) were grown in 96-well plates for 12 h. Then, the cells were treated with 1 μg/mL LPS for 24 h to establish a model of inflammation.

### 4.8. MTT Assay

After RAW264.7 cells were treated with different concentrations (10, 25, 50, 100, 200 μmol/L) of eupatilin for 24 h, the effect of eupatilin on cell viability was monitored with the MTT assay. An MTT solution (5 mg/mL in PBS buffer, 20 μL) was added and incubated with the cells for 4 h. Then, the culture medium was replaced with 150 μL DMSO to dissolve the formed violet crystals. Finally, the absorbance was measured at 490 nm [60].

### 4.9. Measurement of NO

NO content in the cell supernatant was measured using Griess reagent [61]. First, RAW264.7 cells (2 × 10^4^/well) were seeded in 96-well plates for 12 h. Second, RAW264.7 cells were treated with different concentrations (10, 25, 50, 100 μmol/L) of eupatilin and 1 μg/mL LPS for 24 h. After treatment, 50 μL supernatant from each well was mixed with 50 μL Griess Reagent I and 50 μL Griess Reagent II and then incubated for 10 min at room temperature. The optical density was determined at 540 nm.

### 4.10. Measurement of ROS

Intracellular ROS levels were measured using 2′,7′-dichlorofluorescein-diacetate (DCFH-DA) [62]. DCFH-DA is readily oxidized by intracellular ROS to fluorescent DCF. RAW264.7 cells (5 × 10^5^/well) were cultured in 6-well plates for 12 h. Then, cells were treated with different concentrations (10, 25, 50, 100 μmol/L) of eupatilin and 1 μg/mL LPS for 24 h. Following LPS and eupatilin treatment, RAW264.7 cells were incubated with 10 µmol/L DCFH-DA for 20 min at 37 °C and then washed three times with PBS. Finally, the fluorescence was measured under a fluorescence microscope at an excitation wavelength of 488 nm and an emission wavelength of 525 (Nikon Corporation, Tokyo, Japan).

### 4.11. Measurement of IL-6

RAW264.7 cells (2 × 10^4^/well) were cultured in 96-well plates for 12 h. Then, cells were treated with different concentrations (10, 25, 50, 100 μmol/L) of eupatilin and 1 μg/mL LPS for 24 h. After treatment, absorbing cell supernatants, IL-6 levels in cell supernatants were determined using ELISA kits according to the manufacturer’s instructions.

### 4.12. Western Blot Analysis

RAW264.7 cells were seeded in six-well plates at a density of 5 × 10^5^ cells/well. After 24 h of treatment with LPS and eupatilin, the RAW264.7 cells were washed with cold PBS and then lysed in 200 µL RIPA buffer containing phosphatase inhibitors and protease inhibitors for 20 min. An amount of 100 mg lung tissue was added with 1 mL RIPA buffer containing phosphatase inhibitors and protease inhibitors, homogenized, and placed on ice for 20 min.

The lysate was centrifuged at 12,000× *g* rpm and 4 °C for 10 min, and the supernatant was stored at –80 °C. The protein concentration was determined with a BCA protein detection kit. Thirty micrograms of protein was added to each well, separated by 10% SDS–PAGE, and transferred to PVDF membranes. The membranes were blocked with 5% skim milk for 90 min and then incubated with a primary antibody at 4 °C overnight. Then, the membranes were washed with TBST three times and incubated with an appropriate secondary antibody. Then, the bands were visualized using an enhanced chemiluminescence (ECL) kit (Pierce Biotechnology, Rockford, IL, USA). Finally, ImageJ software (NIH, Bethesda, MD, USA) was used for gray integration analysis.

### 4.13. Statistical Analysis

Data are expressed as the mean ± SEM. GraphPad Prism 9.0 software (San Diego, CA, USA) was used for statistical analysis, and one-way ANOVA followed by multiple comparison tests was performed. Statistical significance was accepted at *p* < 0.05.

## Abbreviations

NF-κB	nuclear factor kappa B
MAPKs	mitogen-activated protein kinases
JNK	c-Jun N-terminal kinase
Nrf2	nuclear factor-erythroid 2-related factor 2
OVA	ovalbumin
Dex	dexamethasone
BALF	bronchoalveolar lavage fluid
LPS	lipopolysaccharide
FBS	fetal bovine serum
MTT	3-(4,5-dimethylthiazol-2-yl)-2,5-diphenyl tetrazolium bromide
ROS	reactive oxygen species
